# Genetic variants are identified to increase risk of COVID-19 related mortality from UK Biobank data

**DOI:** 10.1186/s40246-021-00306-7

**Published:** 2021-02-03

**Authors:** Jianchang Hu, Cai Li, Shiying Wang, Ting Li, Heping Zhang

**Affiliations:** Department of Biostatistics, Yale University, 300 George Street, Ste 523, New Haven, CT 06511 USA

**Keywords:** COVID-19, GWAS, Host genetic factors, Mortality, SARS-CoV-2, UK Biobank

## Abstract

**Background:**

The severity of coronavirus disease 2019 (COVID-19) caused by the severe acute respiratory syndrome coronavirus 2 (SARS-CoV-2) is highly heterogeneous. Studies have reported that males and some ethnic groups are at increased risk of death from COVID-19, which implies that individual risk of death might be influenced by host genetic factors.

**Methods:**

In this project, we consider the mortality as the trait of interest and perform a genome-wide association study (GWAS) of data for 1778 infected cases (445 deaths, 25.03%) distributed by the UK Biobank. Traditional GWAS fails to identify any genome-wide significant genetic variants from this dataset. To enhance the power of GWAS and account for possible multi-loci interactions, we adopt the concept of super variant for the detection of genetic factors. A discovery-validation procedure is used for verifying the potential associations.

**Results:**

We find 8 super variants that are consistently identified across multiple replications as susceptibility loci for COVID-19 mortality. The identified risk factors on chromosomes 2, 6, 7, 8, 10, 16, and 17 contain genetic variants and genes related to cilia dysfunctions (*DNAH7* and *CLUAP1*), cardiovascular diseases (*DES* and *SPEG*), thromboembolic disease (*STXBP5*), mitochondrial dysfunctions (*TOMM7*), and innate immune system (*WSB1*). It is noteworthy that *DNAH7* has been reported recently as the most downregulated gene after infecting human bronchial epithelial cells with SARS-CoV-2.

**Conclusions:**

Eight genetic variants are identified to significantly increase the risk of COVID-19 mortality among the patients with white British ancestry. These findings may provide timely clues and potential directions for better understanding the molecular pathogenesis of COVID-19 and the genetic basis of heterogeneous susceptibility, with potential impact on new therapeutic options.

**Supplementary Information:**

The online version contains supplementary material available at 10.1186/s40246-021-00306-7.

## Introduction

Coronavirus disease 2019 (COVID-19) is a highly infectious disease caused by the severe acute respiratory syndrome coronavirus 2 (SARS-CoV-2). The pneumonia was first reported in December 2019 in Wuhan, Hubei Province, China, followed by an outbreak across the country [1, 2]. As of September 8, 2020, the pandemic of COVID-19 has rapidly spread worldwide and caused over 27 million infected cases and 891,000 deaths (3.3%) according to JHU COVID-19 dashboard [3]. Currently, the effective therapeutic measures available to counteract the SARS-CoV-2 are limited. While studies have been dedicated to investigating the clinical features, epidemiological characteristics of COVID-19 [4–11], and genomic characterization of SARS-CoV-2 [12], few are through the lens of statistical genetics and the host genetic factors contributing to COVID-19 remain largely enigmatic [13, 14]. Moreover, the severity of COVID-19 and course of the infection is highly heterogeneous. The majority of COVID-19 cases only have mild or no symptoms, while some of the patients develop serious health outcomes. A UK cross-sectional survey of 20,133 patients who were hospitalized with COVID-19 showed that patients with diabetes, cardiovascular diseases, hypertension, or chronic respiratory diseases were at higher risk of death [15]. More importantly, evidence has shown that males and some ethnic groups have increased risk of death from COVID-19 [16–20]. These observations suggest that there might be host genetic determinants which predispose the subgroup of patients to more severe COVID-19 outcomes. Undoubtedly, there is an urgent need for understanding the host genetic basis of heterogeneous susceptibility to COVID-19 and uncovering genetic risk factors. Current studies mainly focus on investigating associations between host genetic factors and infection or respiratory failure [13, 14]. Obviously, infection may only be partially explained by genetic factors since exposure to the virus could be more important. Here, we consider the mortality as the trait of interest for our analysis.

As of early August 2020, UK Biobank [21, 22] has released the testing results of COVID-19 for 12,428 participants, including 1778 (14.31%) infected cases with 445 deaths related to COVID-19. This dataset accompanied by already available health care data, genetic data, and death data offers a unique resource and timely opportunity for learning the host genetic determinants of COVID-19 susceptibility, severity, and mortality.

In this project, we perform a genome-wide association study (GWAS) exploiting the concept of super variants in statistical genetics to identify potential risk loci contributing to the COVID-19 mortality. A super variant is a combination of alleles in multiple loci in analog to a gene. However, in contrast to a gene that refers to a physically connected region of a chromosome, the loci contributing to a super variant are not restricted by their spatial locations in the genome [23–25]. The rationale behind our analysis is twofold: First, COVID-19 infections require environmental exposure and the genetic contribution may be limited relative to the environmental exposure, while the mortality may have a stronger genetic effect. Second, COVID-19 is a complex syndrome, which may reflect interacting genomic factors, and our analysis based on super variants enables us to leverage gene interactions beyond the additive effects.

## Methods

### Sample processing and genotype quality control

We analyze the COVID-19 data released by UK Biobank (Category ID: 100091) [22] on August 3, 2020, which include in total 1778 of COVID-19 infected cases. Here, we consider an infected case as a sample with any positive PCR test result or a death with virus found. Among infected cases, 445 of them are reported death caused directly or indirectly by COVID-19 and the remainder of 1333 patients are survivors. In our analysis, to limit the potential effect of population structure, we focus on samples from white British ancestry. After standard sample quality controls, there remain 1096 of COVID-19 infected participants, of which 292 are deaths (26.64%) and 804 are survivors. Their imputed genotype data (Field ID: 22801-22822) and clinical variables including gender and age (Field ID: 31, 34) are all accessible from UK Biobank [21].

Our analysis makes use of imputed single-nucleotide polymorphism (SNP) datasets from UK Biobank. SNPs with duplicated names and positions are excluded. After standard genotyping quality control, where variants with low call rate (missing probability ≥ 0.05) and disrupted Hardy-Weinberg equilibrium (*p* value < 1 × 10^−6^) are removed, we retain in total 18,617,478 SNPs. We divide the whole SNP dataset into 2734 non-overlapping local sets according to the physical position so that each set consists of SNPs within a segment of physical length 1 Mbp.

### Statistical analysis

We consider the concept of super variant for GWAS. A super variant is a combination of alleles in multiple loci, but unlike a gene that refers to a physically connected region of chromosome, the loci contributing to a super variant can be anywhere in the genome [24, 25]. The super variant is suggested to be powerful and stable in association studies as it aggregates the strength of individual signals. In addition, it accounts for potential complex interactions between different genes even when they are located remotely. To identify significant super variants, a local ranking and aggregation method is adopted.

**Fig. 1:**
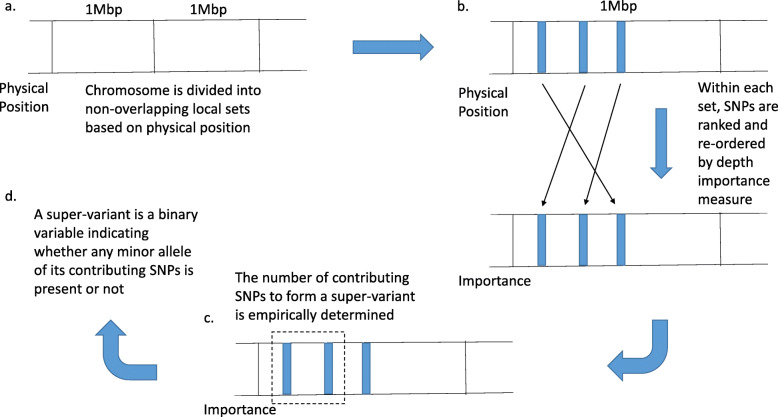
Flow chart of constructing super variants

The method consists of four steps, and a flowchart of the method is presented in Fig. 1. In the first step (Fig. 1a), chromosomes are divided into local SNP sets as described above. In the second step (Fig. 1b), within each set, a tree-based method [26] is utilized to obtain the so-called depth importance measure [27, 28] of each SNP which leads to a ranking of SNPs in terms of their marginal contribution to the mortality. The depth importance measure takes consideration of the effect of a SNP as a splitting node in a classification tree as well as the depth at which it is located in the tree. The rationale behind such a measure is that an important SNP tends to be used in the early stage of the tree growing step. In the third step (Fig. 1c), we empirically determine the number of top SNPs to form a super-variant following [25]. In the last step (Fig. 1d), top SNPs within each local set are then aggregated into a super variant. In addition, two modes of transmission, dominant and recessive modes, are both considered for the super-variant identification. We refer the readers to [25] for more details.

Our analysis considers the following discovery-validation procedure. The complete dataset is randomly divided into two sets, one for discovery and the other for verification. Each set consists of 146 deaths and 402 survivors. We apply the aforementioned ranking and aggregation method for super-variant identification on the discovery dataset. After the discovery of the super variants, we then investigate their associations with the death outcomes of COVID-19 through logistic regression in the verification and complete datasets. Age and gender are considered in the regression analyses as confounders to remove potential bias. We use 1.83 × 10^−5^ (i.e., 0.05/2734) as the threshold for super-variant-level association on the discovery dataset since 2734 SNP sets are considered. A super variant is verified if its logistic regression coefficient achieves the level of 0.05 significance on the verification dataset and super-variant-level significance on the complete dataset.

To ascertain the stability of the associations, we repeat the above procedure 10 times and retain the verified super variants and their contributing SNPs. Typically, genetic association analyses do not include an internal assessment, but we replicate our procedure 10 times as a safeguard strategy for detecting potential and stable signals without dramatically increasing the computational burden. Finally, for super variants that are consistently verified across multiple runs, we conduct Cox regressions with adjustment for age and gender in the complete dataset to further validate their associations.

## Results

We find 216 different verified super variants across 10 repetitions of the discovery-validation procedure. More importantly, there are two super variants, chr6_148 and chr7_23, identified in 4 out of 10 repetitions. In addition, there are 6 super variants, chr2_197, chr2_221, chr8_99, chr10_57, chr16_4, and chr17_26 identified in 3 out of 10 repetitions. According to the binomial distribution, the probability of a super variant being verified in 4 (3) out of 10 repetitions by chance is at most 0.00096 (0.0105) if *p* value in the verification dataset is assumed to be uniformly distributed.

In terms of the SNPs contributing to these 8 super variants, there exist SNPs selected multiple times across different repetitions. Specifically, for chr6_148, SNP rs117928001 is a contributing SNP in all 4 times when this super variant is verified, and there are another 94 contributing SNPs selected 3 times. Similarly, for chr7_23, SNP rs1322746 is a contributing SNP in 3 repetitions when this super variant is verified, and another 4 SNPs are selected 2 times. For super-variant chr2_197 which is identified in 3 out of 10 repetitions, SNPs rs34011564 and rs71040457 are both contributing SNPs in all 3 times. For chr8_99, SNPs rs4735444 and rs531453964 are contributing SNPs of verified super variants in all 3 repetitions. SNPs rs117217714, rs2176724, rs9804218, and rs2301762 are contributing SNPs for chr17_26, chr2_197, chr10_57, and chr16_4 in all 3 repetitions when these super variants are verified, respectively. We calculate minor allele frequency (MAF), odds ratio (OR), and *p* value for the contributing SNPs of the 8 super variants based on the complete dataset. See Table S1 in Additional file 1 for the details of all contributing SNPs which are selected in at least 2 repetitions.

**Fig. 2:**
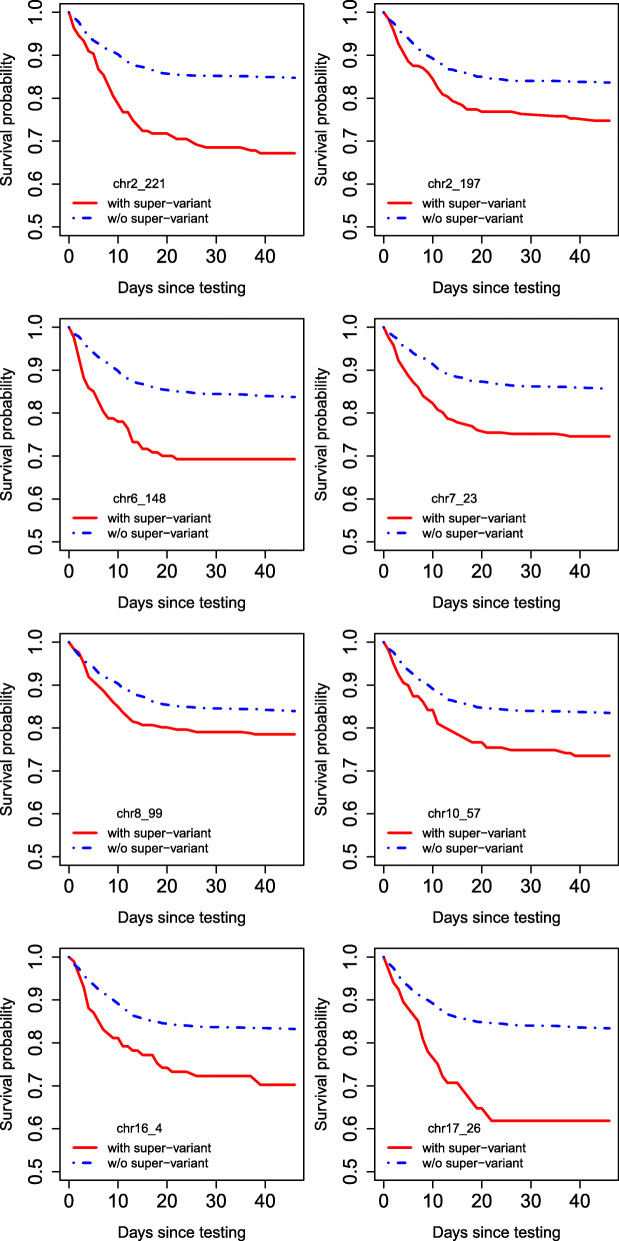
Survival curves of 8 identified super variants in the complete dataset

**Fig. 3:**
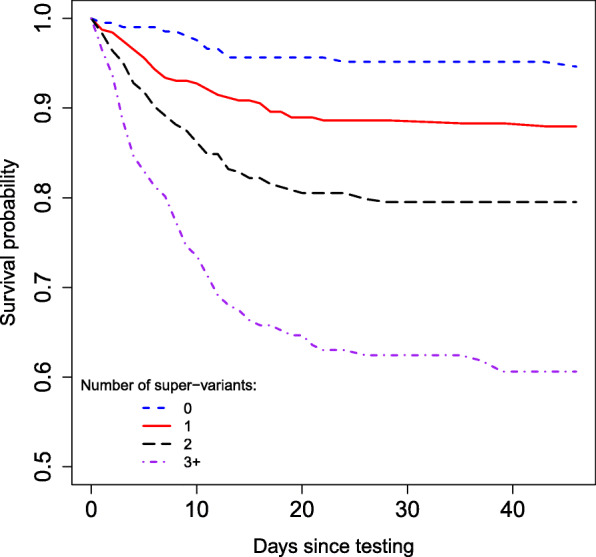
Survival curves stratified by the number of super variants in the complete dataset

We use SNPs which are selected in at least 2 repetitions to representatively form 8 super variants according to the same mode of transmission (dominant/recessive) when they are discovered. Table 1 gives their effects estimated from univariate logistic regression and Cox regression with adjustment for sex and age in the complete dataset. For the logistic regression, all of them achieve super-variant-level significance (i.e., *p* value < 1.83 × 10^−5^). The strongest signal in terms of *p* value is given by chr7_23 (*p* value = 9.5 × 10^−9^), and the largest odds ratio appears at chr17_26 (OR = 4.237). For the Cox regression, the largest individual hazards ratio (HR) appears at chr17_26 (HR = 2.956) as well, and the smallest individual *p* value is given by chr2_221 (*p* value = 5.2 × 10^−9^). Table 2 lists the details of representative contributing SNPs with high selection frequency and important gene mapping results of the 8 super variants. Figure 2 shows that the survival probabilities of the patients with identified super variants remarkably drop during the first 20 days since testing, suggesting of risk genotypes. Figure 3 presents the survival probabilities stratified by the number of super variants. Note that the super variants are weighted equally. The HR of super variants is 1.778 with 95% CI being [1.593, 1.985], and the associated *p* value is 1.1 × 10^−24^, while the *p* values of sex and age are 1.2 × 10^−2^ (HR = 1.489, male) and 2.9 × 10^−18^ (HR = 1.107), respectively. The survival probability of patients with more than 3 super variants dramatically decreases to around 0.6 during the first 3 weeks.

**Table 1:** Marginal effects of 8 super variants in the complete dataset

**Dominant**	**Gene**	**OR**	**95% CI of OR**	***p*** **value**	**HR**	**95% CI of HR**	***p*** **value**
chr6_148	*STXBP5/STXBP5-AS1*	2.909	[1.938, 4.365]	1.4 × 10^-7^	2.048	[1.435, 2.921]	7.7 × 10^-5^
chr8_99	*CPQ*	1.923	[1.419, 2.605]	1.6 × 10^-5^	1.502	[1.119, 2.015]	6.7 × 10^-3^
chr16_4	*CLUAP1*	2.725	[1.744, 4.259]	7.0 × 10^-6^	2.123	[1.433, 3.143]	1.7 × 10^-4^
chr17_26	*WSB1*	4.237	[2.472, 7.263]	8.4 × 10^-8^	2.956	[1.949, 4.482]	3.4 × 10^-7^
**Recessive**	**Gene**	**OR**	**95% CI of OR**	***p*** **value**	**HR**	**95% CI of HR**	***p*** **value**
ch2_197	*DNAH7/SLC39A10*	2.553	[1.801, 3.616]	7.3 × 10^-8^	1.625	[1.170, 2.257]	3.8 × 10^-3^
chr2_221	*DES*/*SPEG*	2.739	[1.893, 3.963]	4.9 × 10^-8^	2.614	[1.894, 3.609]	5.2 × 10^-9^
chr7_23	*TOMM7*	2.411	[1.774, 3.276]	9.5 × 10^-9^	1.943	[1.451, 2.603]	8.1 × 10^-6^
chr10_57	*PCDH15*	2.521	[1.736, 3.662]	7.1 × 10^-7^	1.813	[1.283, 2.561]	7.4 × 10^-4^

**Table 2:** SNPs with high selection frequency and important gene mapping results in 8 super variants

Super variant	Chr	SNP name	Position	Minor allele	Major allele	MAF	OR	***p*** value
chr2_197	2	rs73060484	196364477	C	A	0.069	1.945	6.0 × 10^-4^
		rs77578623	196369073	T	C	0.070	1.939	6.2 × 10^-4^
		rs74417002	196384505	G	A	0.034	1.832	3.0 × 10^-2^
		rs73070529	196412097	A	C	0.048	2.249	3.6 × 10^-4^
		rs113892140	196439005	A	G	0.044	2.031	2.8 × 10^-3^
		rs200008298	196602155	AATACT	A	0.032	1.8	3.1 × 10^-2^
		rs183712207	196611282	A	G	0.007	4.783	7.7 × 10^-3^
		rs191631470	196859045	T	C	0.007	3.335	3.9 × 10^-2^
		rs2176724	196952410	A	G	0.138	1.484	6.1 × 10^-3^
chr2_221	2	rs71040457	220294782	A	AG	0.355	1.331	7.7 × 10^-3^
chr6_148	6	rs117928001	147514999	T	C	0.049	2.749	1.1x10^-5^
		rs116898161	147538692	G	A	0.046	2.541	6.9 × 10^-5^
chr7_23	7	rs13227460	22588381	T	C	0.278	1.3	2.6 × 10^-2^
		rs55986907	22817292	T	C	0.286	1.601	3.5 × 10^-5^
chr8_99	8	rs7817272	98140470	C	T	0.194	1.736	1.7 × 10^-5^
		rs4735444	98140991	T	C	0.201	1.784	5.8 × 10^-6^
		rs1431889	98141643	C	G	0.193	1.704	3.5x10^-5^
		rs2874140	98142930	T	A	0.194	1.694	4.0 × 10^-5^
		rs531453964	98143128	CA	C	0.185	1.849	3.2x10^-6^
		rs7007951	98146644	T	C	0.184	1.711	4.4x10^-5^
		rs920576	98147539	C	T	0.201	1.615	1.6x10^-4^
chr10_57	10	rs9804218	56495374	G	C	0.357	1.373	3.3x10^-3^
chr16_4	16	rs2301762	3550977	G	C	0.055	2.541	2.0x10^-5^
chr17_26	17	rs60811869	25590833	C	T	0.024	2.966	6.5x10^-4^
		rs117217714	25987181	C	T	0.013	6.255	3.3x10^-5^

In addition, we use a chi-square test for independence to investigate whether there are any gender differences among the distribution of these 8 super variants as well as differences among distribution of contributing SNPs. For super variants, chr2_197 has *p* value 0.0579 when all samples are considered. The frequency of presenting this super variant among males and females is 18.09% and 22.93%, respectively. For contributing SNPs, rs4346407 on chromosome 2 has *p* value 0.050 when all samples are considered, and SNP 10:56525802_CT_C has *p* value 0.0078 when only death cases are considered. The distributions of these two SNPs are given in Table 3.

**Table 3:** Allelic distribution of contributing SNPs

**rs4346407**	**0**	**1**	**2**
Female	218	227	45
Male	236	255	80
**10:56525802_CT_C**	**0**	**1**	**2**
Female	76	21	9
Male	101	68	13

## Discussion

**Fig. 4:**
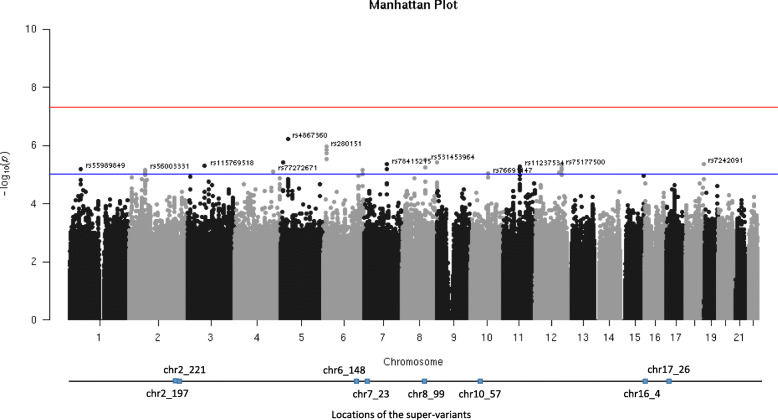
Manhattan plot of traditional single SNP association analysis based on samples with white British ancestry only and controlled for gender and age. The red horizontal line corresponds to the commonly adopted genome-wide significant level at 5 × 10^−8^, and the blue horizontal line represents the suggestive significant level at 1 × 10^−5^. Top SNPs above the suggestive line in each chromosome and the eight identified super-variant regions are annotated

As the COVID-19 pandemic creates a global crisis of overwhelming morbidity and mortality, it is urgent and imperative to provide insights into how host genetic factors link to clinical outcomes. With the timely release of the UK Biobank COVID-19 dataset, we perform a GWAS for detecting genetic risk factors for COVID-19 mortality. However, due to the limited sample size, the traditional single SNP GWAS has low power in signal detection which is evidenced by the Manhattan plot shown in Fig. 4. This traditional association analysis is also conducted on the same samples with white British ancestry and controlled for gender and age. As demonstrated, the traditional single SNP analysis method is unable to detect any genome-wide significant association with commonly used threshold 5 × 10^−8^, which motivates us to consider the concept of super variant for GWAS.

From Table 1, we can see that the magnitudes of the odds ratios calculated for the identified super variants tend to be larger than those of the traditional GWAS signals. This might be because some of the super variants contain rare variants (low MAF) as the contributing SNPs, and it is known that rare variants with large effect sizes and common variants with small effect sizes are relatively easier to identify using GWAS [29]. Indeed, among the eight identified super variants, half of them contain rare variants with MAF less than 0.05, one super-variant chr16_4 contains a variant with MAF equal to 0.055. Moreover, the top two super variants (chr17_26 and chr6_148) with the largest magnitude of the odds ratio all consist of rare variants. However, in a previous research of associating super variants and breast cancer [25], the magnitudes of the odds ratios for the super variants were found to be comparable to those of the single SNP variants. Therefore, this phenomenon appears to be study and disease dependent.

Although the identified super variants are similarly distributed in males and females, the results presented in Table 3 suggest that males tend to present more minor alleles for two contributing SNPs rs4346407 and 10:56525802_CT_C which potentially increase their risk of COVID-19 mortality. Such a phenomenon of higher risk for males has been reported in recent studies [17, 18, 30, 31].

The identified super variants are mapped to annotated genes. The most interesting signal appears on chromosome 2 in the super-variant chr2_197. Within this super variant, SNPs rs183712207 and rs191631470 are located in the intron of gene *DNAH7*, and SNP rs200008298 is located in the downstream of gene *DNAH7* (distance = 271 bp). Using Combined Annotation Dependent Depletion (CADD) tool [32], we find that SNP rs200008298 has scaled C-score = 14.42, which means the variant is predicted to be the 14% most deleterious substitution in the human genome. Gene *DNAH7* encodes dynein axonemal heavy chain 7, which is a component of the inner dynein arm of ciliary axonemes [33]. A recently published paper showed that gene *DNAH7* is the most downregulated gene after infecting human bronchial epithelial cells with SARS-CoV2 [34]. The authors of that study speculated that the downregulation of gene *DNAH7* causes the reduction of function of respiratory cilia. Our results suggest that COVID-19 patients with variations in gene *DNAH7* may have higher risk for dying from COVID-19. In addition, within the super-variant chr2_197, SNPs rs200008298 (3 prime UTR), rs4578880 (intron), and rs113892140 (upstream) are related to gene *SLC39A10* which encodes a zinc transporter. This gene has been reported to facilitate antiapoptotic signaling during early B cell development [35], modulate B cell receptor signal strength [36], and control macrophage survival [37].

Signal at super-variant chr16_4 is also related to cilia. This super variant consists of a single SNP rs2301762, which is located in 5 prime UTR of gene *CLUAP1* and it belongs to promotor region. Gene *CLUAP1* encodes clusterin-associated protein 1 [38, 39], which is an evolutionarily conserved protein required for ciliogenesis [40].

Chr2_221 consists of 3 SNPs. SNP rs71040457 is located in the downstream of gene *DES* (distance = 3322 bp) and the upstream of gene *SPEG* (distance = 4917 bp). Mutations in both gene *DES* and *SPEG* are reported to be associated with cardiomyopathy [41–43]. Several studies have reported cardiomyopathy in COVID-19 patients [44, 45], and acute myocardial damage caused by SARS-CoV-2 greatly increases the difficulty and complexity of patient treatment [46].

Chr7_23 is composed of five intergenic variant SNPs. Among them, SNP rs55986907 is an expression quantitative trait locus (eQTL) of gene *TOMM7* in multiple tissues according to the Genotype-Tissue Expression (GTEx) database. This SNP has a scaled C-score = 12.01 from CADD tool. The gene product of *TOMM7* is a subunit of the translocase of the outer mitochondrial membrane, and plays a role in regulating the assembly and stability of the translocase complex [47].

Super-variant chr6_148 contains 101 SNPs. Eighty-nine of them are located in the intron of gene *STXBP5* and six of them are located in the intron of gene *STXBP5-AS1*. On the one hand, a study showed that gene *STXBP5* inhibits endothelial exocytosis and promotes platelet secretion, and the variation within *STXBP5* is a genetic risk for venous thromboembolic disease [48]. On the other hand, studies have revealed that *STXBP5-AS1* encodes a long noncoding RNA, which inhibits cell proliferation, migration, and invasion via preventing the phosphatidylinositol 3 kinase/protein kinase B (PI3K/AKT) signaling pathway against *STXBP5* expression in non-small-cell lung carcinoma and gastric cancer cells [49, 50]. Our results suggest that the variations within *STXBP5*/*STXBP5-AS1* and the interaction between them may result in increased risk of death among COVID-19 patients through the mechanism related to endothelial exocytosis.

Chr17_26 is composed of three intergenic variant SNPs. Among them, SNP rs60811869 is an eQTL of gene *WSB1* in artery-tibial tissue based on the GTEx database. This gene has been reported to function as a Interleukin-21 (IL-21) receptor binding molecule, which enhances the maturation of IL-21 receptor [51].

Super-variant chr10_57 contains 11 SNPs and all of them are located in the intron of gene *PCDH15*. Gene *PCDH15* is essential for maintenance of normal retinal and cochlear function.

Super-variant chr8_99 is composed of 7 SNPs. All the SNPs are located in the intron of gene *CPQ*. Among them, SNPs rs7817272 and rs1431889 have scaled CADD scores larger than 10. Gene ontology (GO) annotations of this gene include protein homodimerization activity and carboxypeptidase activity.

There are multiple limitations and future directions to our study and analysis results. First, the roles of the identified super variants and related genes in COVID-19 susceptibility are not substantiated by functional validation. Nevertheless, our results warrant future investigation to learn the relationship between genetic variations and the severe COVID-19 outcomes. Second, preexisting comorbidities may represent important risk factors for COVID-19 vulnerability. A recent study showed that the most common comorbidities in hospitalized COVID-19 patients from UK Biobank were hypertension, fragility fractures, coronary heart disease, type 2 diabetes, and asthma. However, among these preexisting conditions, only type 2 diabetes was identified as significant for related mortality [52]. As a first attempt in identifying potential host genetic risk factors associating with COVID-19 mortality, it is reasonable for us to focus on genetic signals given the complexity of the preexisting conditions, but incorporating such information into association studies is a valid direction. Third, the impact of social and economic disparities on COVID-19 susceptibility has been well documented [53, 54]. Although our study aims to identify genetic risk factors for COVID-19 mortality, further research is needed to determine how genetic factors may interact with environmental factors that influence access to high-quality health care. Fourth, our study is restricted by the limited sample size. We anticipate a continuous accumulation of data in the following months and plan to iterate our analysis whenever more data become available. Last but not the least, we currently focus on the population with white British ancestry of UK Biobank in the analysis, validating the identified risk factors in independent populations from other resources or ethnic groups worth further investigation.

## Conclusions

We identify 8 potential genetic risk loci for the mortality of COVID-19. These findings may provide timely clues and potential directions for better understanding the molecular pathogenesis of COVID-19 and genetic basis of heterogeneous susceptibility, with potential impact on new therapeutic options.

## Supplementary Information

**Additional file 1: Table S1.** SNPs corresponding to 8 super-variants. Statistics are based on complete dataset.

## Data Availability

The data used in the study are available with the permission of the UK Biobank (https://www.ukbiobank.ac.uk).
